# Oncolytic viruses in head and neck cancer: therapeutic promise and translational challenges

**DOI:** 10.3389/fimmu.2026.1889008

**Published:** 2026-08-28

**Authors:** Haiting Wang, Hui Shen, Mingjun Zhang, Yakui Mou, Chao Ren, Yu Sun, Wanchen Liu, Caiyu Sun, Dongxian Li, Yumei Li, Peng Qu, Xicheng Song

**Affiliations:** 1 Shandong Provincial Clinical Research Center for Otorhinolaryngologic Diseases, Yantai Yuhuangding Hospital, Qingdao University, Yantai, China; 2 Department of Otorhinolaryngology, Head and Neck Surgery, Yantai Yuhuangding Hospital, Qingdao University, Yantai, Shandong, China; 3 Shandong Provincial Key Laboratory of Neuroimmune Interaction and Regulation, Yantai, Shandong, China; 4 Yantai Key Laboratory of Otorhinolaryngologic Diseases, Yantai, Shandong, China

**Keywords:** clinical application, combination therapy, head and neck squamous cell carcinoma, oncolytic virotherapy, oncolytic virus

## Abstract

Head and neck squamous cell carcinoma (HNSCC) remains difficult to treat, particularly in patients with HPV-negative disease. These patients often get diagnosed too late, their cancer doesn’t respond well to treatment, and they don’t live as long. Oncolytic viruses (OVs) can be used for treating cancer instead of using other cytotoxic methods since they can kill malignant cells directly and cause both local and systemic anti-cancer immune responses. In this review, we focus on current evidence of oncolytic virotherapy (OVT) in HNSCC, including that OVs selectively infect tumor cells, induce tumor cell lysis and immunogenic cell death, and modulate the tumor microenvironment. We also compare some OV platforms which were studied in HNSCC or similar solid-tumor settings: Herpes simplex virus (HSV), adenovirus, vaccinia virus, reovirus, measles virus, vesicular stomatitis virus, and Newcastle disease virus. Finally, we discuss emerging strategies to improve the efficacy and clinical translation of OVT for HNSCC, including tumor- or stimulus-responsive promoters, engineering of viral tropism and receptor specificity, modulation of both antiviral and immunosuppressive pathways, and incorporation between therapeutic payloads and advanced delivery systems. Altogether these developments support further exploration of OVs within biomarker-informed multimodal therapy for select patients of HNSCC.

## 1 Introduction

Head and neck squamous cell carcinoma (HNSCC) includes various kinds of malignant epithelial tumors which mostly occur in the oral cavity, oropharynx, hypopharynx, larynx and other mucosal areas. It is still a big problem all over the world. GLOBOCAN 2022: head and neck cancers make up roughly 950,000 new cases and nearly 480,000 deaths yearly; that’s around 5% of global cancer load (1). The incidence differs greatly across different geographical regions. It is also strongly associated with tobacco use, alcohol drinking, betel nut chewing, and human papillomavirus (HPV) infection (2). In many developing places, people keep getting exposed to things like tobacco, alcohol, and betel nuts, which has led to more sicknesses (3).

HPV-negative HNSCC is still hard to deal with. Compared to HPV-positive diseases, HPV-negative tumors tend to be more commonly found in smokers and drinkers who have had many changes to their TP53 gene, a weak immune system, and an even worse place for cancer cells to grow called TME (2–5). These biological features partly explain their lower sensitivity to radiotherapy and immune checkpoint blockade.

Another difficulty is that many people get sick only when it’s already quite bad. The first symptoms are usually not very clear and there can be primary tumors at complicated places, so about 60% of patients have locally advanced disease on their first visit to see a doctor (6–8). Even after using multiple modes of treatment such as surgery, radiotherapy, chemotherapy and immunotherapy, the 5-year overall survival rate for advanced HNSCC is still unsatisfactory, especially in those patients who have recurrence, metastasis or resistant to therapy (8).

Therefore, OVT is now considered an add-on to HNSCC therapy. Unlike normal cytotoxic treatments, oncolytic viruses could both kill tumor cells and promote antitumor immunity in the TME at the same time. Talimogene laherparepvec (T-VEC), ONYX-015, Reolysin etc., several OVs have been assessed through clinical or translation trials regarding HNSCC, offering grounds for further research into OV-based monotherapy and combination treatment.

Over 100 years ago, viruses were used as experimental agents that would cause cell death or dysfunction of cancer cells. The concept that viruses could selectively kill tumor cells was proposed by researchers in the 1990s, leading to the definition of OVs (9). OVs are either natural or genetically modified viruses that selectively infect and lyse cancer cells while sparing normal tissues, often stimulating the anti-tumor immune response.

The field of OVT was rapidly enhanced in the late 20th century with the development of cell culture techniques and molecular biology tools (10). Most OVs today are genetically engineered to increase their ability to infect tumor cells selectively while minimizing toxicity to normal cells (11).

OVs are generally categorized into three generations: First-generation OVs are naturally occurring viruses which replicate preferentially in tumor cells without genetic modifications. Second-generation OVs are viruses which are engineered to enhance tumor selectivity and reduce toxicity through genetic modifications. In 2015, Talimogene laherparepvec (T-VEC or Imlygic), one second-generation recombinant HSV-1 engineered to express granulocyte-macrophage colony-stimulating factor (GM-CSF), became the first OV approved by the U.S. Food and Drug Administration (FDA) (12). Third-generation OVs are further genetically modified to express immune-stimulatory genes, enhance anti-tumor immunity, and improve tumor specificity.

The review provides an overview of OVT in HNSCC, identifies key limitations in clinical translation, and outlines engineering strategies aimed at improving efficacy, safety, and patient selection. We also discuss OV combination therapies with existing therapies to overcome immune evasion and enhance efficacy.

## 2 Mechanisms of OVs

**Figure 1:**
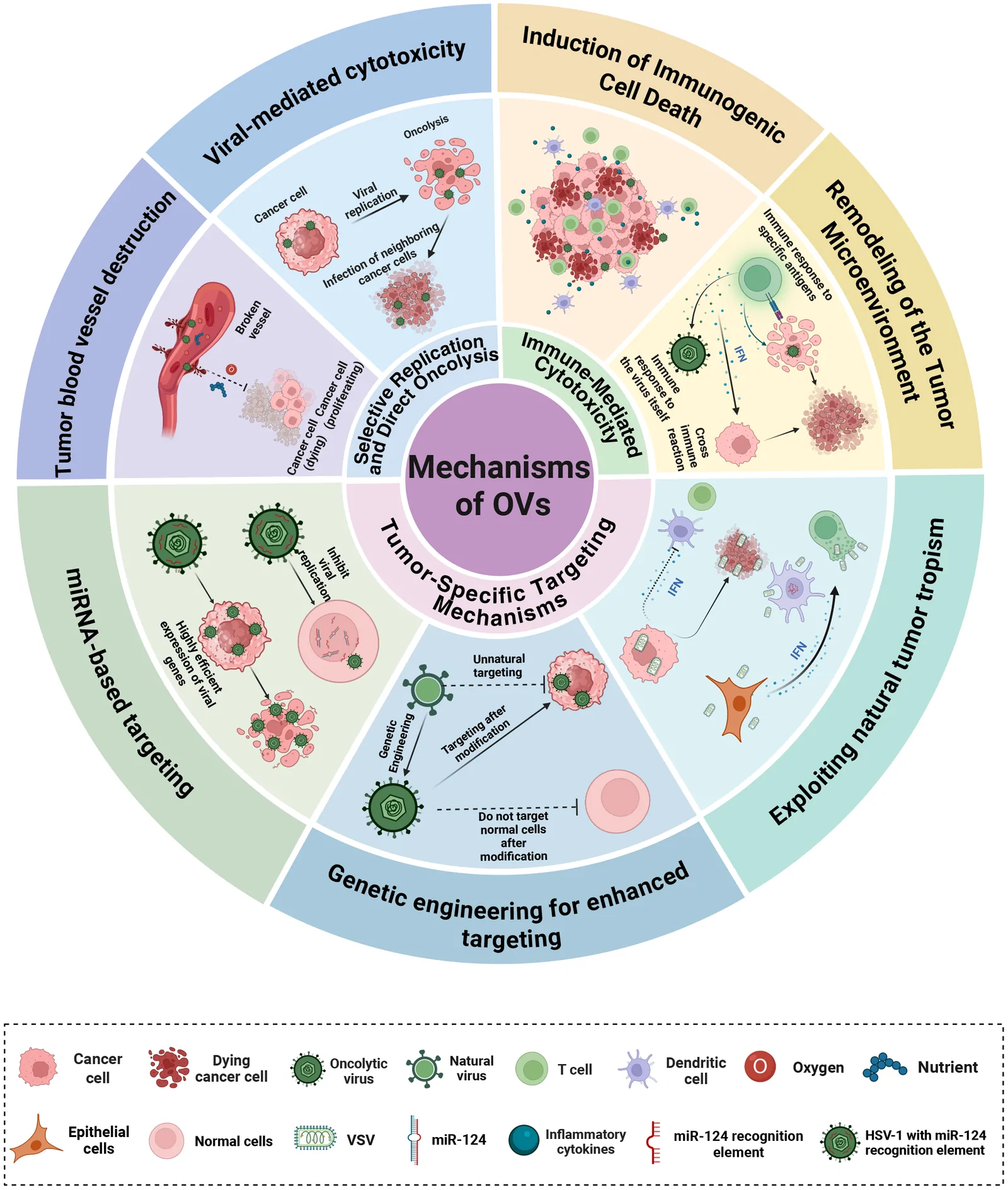
The mechanism of OVT mediates tumor regression. The mechanism of OVT involves several pathways that collectively mediate tumor regression. By harnessing these pathways, OVs therapy significantly enhances tumor regression, presenting a promising therapeutic strategy for cancer treatment. The figure was created using BioRender (https://biorender.com/).

OVs exert anti-cancer effects through the combination of direct tumor cell lysis and immune modulation. Unlike traditional cytotoxic therapies, OVs offer a multifaceted mechanism of action that leverages both virology and tumor immunology. Their therapeutic efficacy arises from five interconnected processes (**Figure 1**).

### 2.1 Tumor-selective replication and oncolysis

OVs infect cancer cells via receptors that are often highly expressed on tumor cells and utilize the resources within these tumor cells to replicate, disrupting the metabolism and physiological functions, ultimately leading to cell death-–-the process is known as oncolysis (9). Most OVs are engineered to exploit the molecular vulnerabilities of tumor cells, such as dysregulated interferon (IFN) signaling, defective antiviral responses, and over-expression of viral entry receptors. Once inside a cancer cell, OVs hijack the replication machinery of the host cell, resulting in rapid viral amplification, cellular stress, and eventual lysis. The direct cytolytic effect reduces tumor burden, and creates a pro-inflammatory milieu enriched with pathogen-associated molecular patterns (PAMPs) and damage-associated molecular patterns (DAMPs), such as ATP, HMGB1, and calreticulin (11, 13, 14).

### 2.2 Induction of immunogenic cell death

OV-mediated lysis often initiates ICD, a form of cell death that recruits and activates innate immune cells, particularly antigen-presenting cells (APCs) such as dendritic cells (DCs) (15). Through cross-presentation of tumor-associated antigens released from lysed cells, OVs initiate a robust adaptive immune response. The process converts immunologically “cold” tumors which are characterized by T-cell exclusion and antigen ignorance into “hot” tumors that are primed for immune attack (11, 14).

### 2.3 Remodeling of the tumor microenvironment

OVs can alter the tumor microenvironment through both viral replication and the immune response triggered by infected tumor cells. Viral infection promotes the release of tumor antigens, pathogen-associated molecular patterns, and damage-associated molecular patterns, which can recruit and activate antigen-presenting cells and effector lymphocytes. In some settings, OV treatment also reduces immunosuppressive components within the TME, including regulatory T cells, myeloid-derived suppressor cells, and inhibitory cytokine networks. To reinforce this effect, some OV platforms were made with immune-stimulatory cytokines such as GM-CSF, IL-2 and IL-12, or matrix-modifying enzymes like PH20. These modifications may improve viral spread, increase immune-cell infiltration, and enhance the recruitment or activation of cytotoxic T lymphocytes and natural killer cells (**Figure 1**).

### 2.4 Genetic engineering for enhanced immune engagement

Genetic engineering has become a main way to make OVs more effective against tumors. Apart from genes which increase tumor selectivity or viral replication, many current OV designs have therapeutic payloads that work directly on the immune microenvironment. These payloads include immune checkpoint inhibitors, bispecific T-cell engagers, and costimulatory molecules such as OX40L and CD40L. Delivering these agents locally in tumors could allow OVs to bypass immune suppression without exposing the whole body. Large DNA viruses like HSV-1 and adenovirus are good for this method because they can accommodate relatively large or multiple transgenes (11).

### 2.5 Tumor-specific targeting strategies

To minimize off-target effects and increase the precision of treatment, OVs have been retargeted with transcriptional targeting by using tumor-specific promoters (e.g., E2F1, AFP, hTERT) to limit replication (**Figure 1**). OV replication can be restricted if we incorporate miRNA target sites that are more common in normal tissue than in cancerous tissue like miR-124 so it would stop replicating in healthy cells. Virus enveloped proteins are modified for better affinity towards tumor rich receptors such as EGFR, CD46 and ICAM-1 (16, 17).

These mechanisms establish OVs as unique immunotherapeutic agents capable of both tumor debulking and immune priming, since the two anti-tumor roles are rarely achieved by a single modality (**Figure 1**). The following sections detail how these mechanisms are harnessed across diverse OV platforms currently under investigation for HNSCC.

## 3 OV Platforms for HNSCC: comparative insights

A growing number of OV platforms have entered both preclinical and clinical development for HNSCC. These platforms differ in their genetic architecture, tumor tropism, immunogenicity, and safety profiles. Importantly, their suitability for HNSCC depends on both their ability to induce cytolysis and their capacity to reshape TME, enhancing anti-tumor responses. Thus, we summarize the leading viral platforms under investigation for HNSCC and evaluate their relative strengths and limitations (**Table 1**).

**Table 1:** The diversity and characteristics of OVs.

Virus	Genetic material	Advantages	Disadvantages
HSV	dsDNA, exceeding 150 kb	Wide host range, high infectivity; large genome for transgene insertion; easily modifiable for multiple transgenes.	Limited route of administration; risk of inflammatory response; premature viral clearance.
Adenovirus	Single linear dsDNA, 30–40 kb	High transfection efficiency, safety, and ease of preparing high-titer viruses for clinical treatment.	When administered systemically, there is nonspecific liver accumulation
VACA	dsDNA, 190 kb	Naturally selective for tumor systemic administration, wide host range and replicate rapidly	Serious adverse reactions in some cases
Reovirus	dsRNA, 23.5 kb	High safety and high specificity.	Neurotropic viruses, adverse effects on the central nervous system
MV	ssRNA, 16 kb	High safety, no dose-limiting toxicity; easy to get.	Easily develop drug resistance to the virus to damages the activity of the virus
VSV	ssRNA, 11 kb	High safety; broad tropism, high lytic activity, rapid replication and easy genetic manipulation; mediates cell apoptosis by inducing endoplasmic reticulum stress	Introduce live viruses into certain areas can have impacts on wildlife
NDV	ssRNA, 15 kb	Highly safe; little impact on host DNA	Genome is too small to increases the difficulty of transformation

### 3.1 Herpes simplex virus

Herpes simplex virus (HSV), an enveloped virus with dsDNA protected by the nucleocapsid, and surrounded by the tegument, has two specific serotypes (HSV-1 and HSV-2). HSV can be genetically engineered to selectively replicate in tumor cells while sparing normal tissues as OVs (18). Compared to other viruses, HSV possesses several advantages for cancer therapy: (1) Broad host range and high infection efficiency; (2) The insertion of therapeutic transgene is allowed into its large genome; (3) Flexibility for genetic modification, enabling the incorporation of multiple transgenes (11, 19). Currently, HSV-1 is one of the most commonly used strains of OVs. Several recombinant HSV-1 strains, including T-VEC, R849, G47Δ and HF10, have been developed for HNSCC treatment (**Table 2**).

**Table 2:** The preclinical experiments of HNSCC.

Virus species	Virus name	Year	Country approved	Modification or combination therapy
HSV-1	G207	1999	USA	γ34.5 and ICP6 (UL39) genes (deleted).
	HF10	2006	Japan	Glycoproteins and UL56 (mutated)
	RH2(R849/HF recombinant)	2008	Japan	GB, GD (Mutations), γ34.5 (deleted), R849 and HF recombinant
	T-Vec	2010	USA	γ34.5 and ICP47 (US12) (deleted), GM-CSF (inserted).
	G47Δ	2011	Japan	γ34.5, ICP6 (UL39), and ICP47 (US12)(deleted)
Adenovirus	Gendicine	2003	China	p53 (Contained)
	H103	2003	China	HSP70 (expressed); CMV promoter (replaced); HSP70, IRES, E1a, and poly A (inserted)
	H101	2005	China	E1B-55K (deleted) and viral E3 with four deletions
	KH901	2005	China	GM – CSF (expressed)
	lp11w	2009	USA	E1B - 19K region (mutation)
	5/3Δ24	2016	USA	CR2 region of E1A (24-base pair deletion).
	5/3CB016	2016	USA	CR2 region of E1A (24-base pair deletion) and CR1 region (deletion)
	CAd12-PDL1	2017	USA	PD-L1 and IL-12p70 (inserted)
	TILT-123	2022	Finland	E1 region (deleted), TNFα and IL - 2 (inserted), and the 24-base pair (deleted).
	Ad - MK - siEGFR	2022	Japan	Midkine promoter (regulate) and expression cassette of siRNA (inserted)
	HAdV-5-ΔCAR-FiberAffilin	2023	Germany	E1 region (deleted) and CAR (ablated), Affilin (conjugated).
	OBP-401	–	Japan	OBP-301plus GFP.
	OV1195	–	USA	Fiber knob region of Ad5 (replaced); E1a promoter (replaced); RGD targeting motif/SV40 signal site/hGM – CSF (inserted).
	Ad-TIMP-2	–	USA	TIMP–2 (inserted)
	OBP-301	–	Japan	hTERT promoter (inserted)
VACA	VVleptin	2019	Multiple	Leptin(inserted)
	VVΔTKΔN1L	2020	Multiple	N1L gene (deleted), RFP marker and DT gene (inserted); IL-12 gene (inserted).
	VVΔTKΔN1L - IL12	2020	Multiple	IL-12 gene (inserted).
	VVdnTGFβmm	2023	USA	dnTGFβmm (expressed)
	VV - GFP	2024	USA	GFP gene (inserted)
	GLV - 1h68	–	Multiple	F14.5L, thymidine kinase and hemagglutinin encoding gene(deleted); β-gal/β -glucuronidase genes (inserted).
	VVLister	–	UK, China	Angiostatin or the Escherichia coli lacZ gene (inserted).
Respirovirus	rSeV	2019	Japan	M gene (deleted), GFS (expressed) and uPA2 gene (inserted)
	MV-CD/UPRT-antiEGFR	–	Germany	scFv (inserted) and CD/UPRT gene (inserted)
	MV-CEA	–	–	Soluble extracellular N-terminal domain of CEA
	MV-NIS	–	–	NIS gene (inserted)
Reolysin	Reolysin	2017	USA	Combined with histone deacetylase inhibitors (HDACis).
	Oncolytic Reovirus Type 3	–	Canada	Natural wild-type virus
VSV	rVSV-F	2004	USA	L289A gene (inserted)
	VSV-IFN-β	–	USA	IFN-β gene (inserted)
NDV	NDV(F3aa)-GFP	2006	USA	F protein was replaced with three amino acid changes; GFP gene (inserted).

T-VEC, HSV-1 deficient in neurovirulence factor (γ34.5), leads to tumor-selective replication. The bio-function of γ34.5 is to block the shut-off of protein synthesis and interferon responses in host cells during virus infection. Therefore, the γ34.5 deficient HSV-1 is more sensitive to the above antiviral responses in normal cells. Since tumor cells are often deficient in such host response mechanisms, the γ34.5-deficient virus such as T-VEC can selectively replicate in cancer cells, including Melanoma and HNSCC (20). Furthermore, GM-CSF expression promotes APC activation and T-cell priming (21). R849, another γ34.5-deficient HSV-1 strain, is engineered to express the LacZ marker gene. RH2, derived from R849 and the naturally occurring HF10 strain, lacks γ34.5 and carries mutations in glycoproteins B (gB) and D (gD), which promote syncytium formation and enhance cytotoxicity against oral squamous cell carcinoma (SCC) (22).

G47Δ is a triple mutant strain (deficient γ34.5, ICP6, and ICP47) that has shown efficacy against nasopharyngeal carcinoma (23). The ICP6 deletion enhances sensitivity to acyclovir and ganciclovir, improving clinical safety. The deletion of ICP47 upregulates MHC I presentation, stimulating T lymphocyte activity while reducing natural killer (NK) cell-mediated viral clearance (22).

HF10, a naturally occurring HSV-1 mutant, has deletions in UL43, UL49.5, UL55, UL56, and latent-associated transcripts (LAT), with upregulated UL53 and UL54 expression (24). A Phase I clinical trial in patients with recurrent metastatic HNSCC confirmed that HF10 exhibited anti-metastasis ability (25).

**Figure 2:**
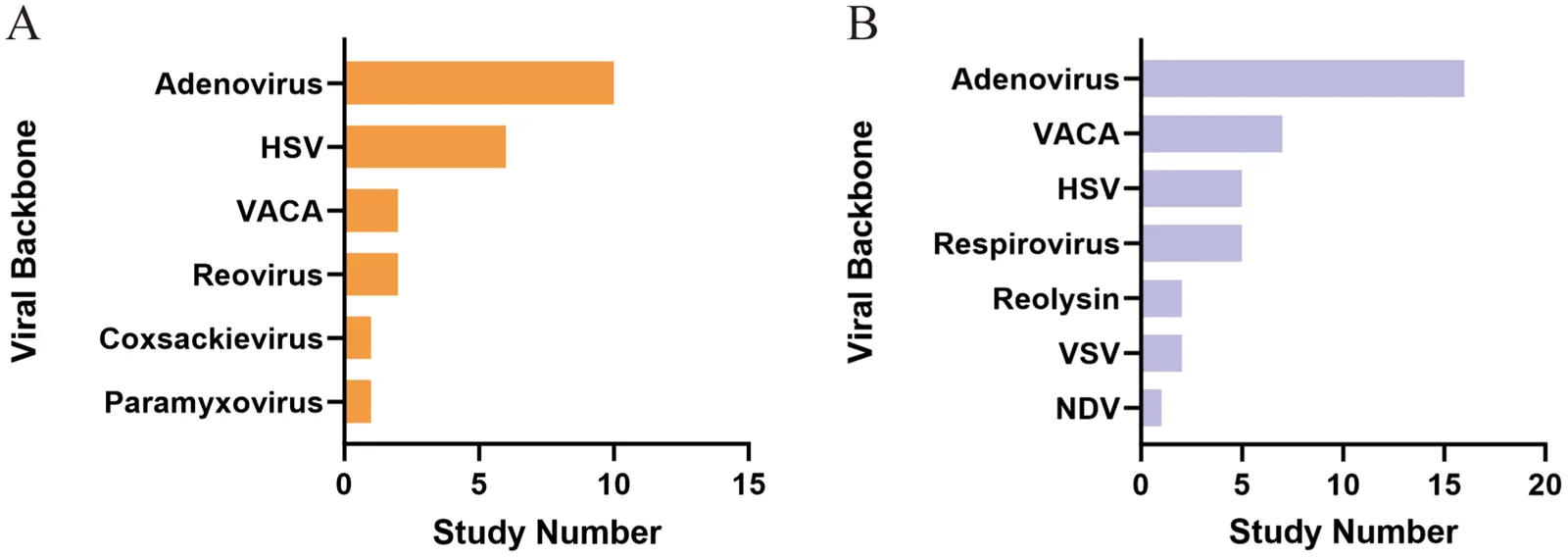
Types of OVs in both clinical trials **(A)** and pre-clinical experiments **(B)** of HNSCC. Types of OVs were reported in clinical trials and preclinical experiments of HNSCC from 1999 to 2024. Among the results, adenovirus is the most dominant viral backbone.

Even though engineered HSV-1 exhibited the ability to replicate efficiently and induced tumor cell death without significant toxicity, preexisting immunity to HSV-1 may reduce systemic efficacy and risk of neurotoxicity in immunocompromised patients, indicating that HSV-1 as an OV needs further engineering (**Figure 2**).

### 3.2 Adenovirus

Adenovirus is a double-stranded, non-enveloped DNA virus is one of the first engineered viruses tested in clinical trials (26). Adenovirus as an OV exhibits high transfection efficiency, enabling effective delivery of therapeutic genes to tumor cells, and allows for easy production of high-titer viruses to meet clinical treatment needs (27). Ads have a low risk of insertion mutagenesis, providing higher safety, even though Ads exert limited genomic capacity (~36 kb) (28).

Different adenoviral mutants exerted varying therapeutic efficacy against HNSCC. A study comparing two AdV type 5 (AdV5) mutants, lp11w (E1B-19K deletion) and lp11w/Δ55k (E1B-19K and E1B-55K deletions), with wild-type AdV5 showed that two mutants induced apoptosis in HNSCC cells by down-regulating cell survival proteins and epidermal growth factor receptor (EGFR) (29). Both mutants exhibited more effective tumor suppression compared to wild-type AdV5 (29). Ad-MK-siEGFR, a recombinant Ads targeting the cytokine Midkine, exhibits strong anti-metastatic effects in HNSCC models (30).

Human papillomavirus (HPV) is a major driver of HNSCC (4). The conditionally replicating adenoviruses (CRAd) leverage the oncogenic functions of HPV E6 and E7 to achieve tumor-selective replication. Deletion mutations within the adenoviral E1A region, such as Δ24, abrogate Rb binding and thus restrict replication in normal cells. In HPV-positive tumors, functional complementation by E7-mediated Rb inactivation and E6-driven p53 suppression restores the viral life cycle, enabling selective lysis of malignant cells. CB016, one type of CRAd, was found to replicate only in HPV-positive HNSCC cells, where E6/E7 downregulate cell cycle checkpoints (31) (**Figure 2**).

### 3.3 Poxvirus

Cowpox virus (CPXV), a member of the Poxviridae family, has a long history of safe use as a smallpox vaccine vector in humans (32). Among the poxviruses, vaccinia virus (VACV), myxoma virus (MV), raccoonpox virus, and Tanapox virus were found to show varying degrees of oncolytic efficacy across different cancer cell lines, with VACV displaying the broadest and most potent activity (32). In HPV-positive HNSCC, VACV has been revealed to enhance anti-tumor immunity by infecting Tregs and exhausted T cells (Tex) within the TME, leading to their depletion and relieving immunosuppression (33).

### 3.4 Reovirus

Reovirus is a non-enveloped, double-stranded RNA virus (34). Its oncolytic potential is closely linked to aberrant RAS signaling (a common mutation in many cancers), by which both viral replication and release are promoted (35, 36). Reovirus typically causes only mild symptoms in humans; however, it selectively replicates in malignant cells, demonstrating potent oncolytic activity (34, 37). Further, HPV-negative cells were found to be more susceptible to reovirus infection and lysis, highlighting reovirus as a promising agent particularly for HPV-negative HNSCC (38) (**Figure 2**).

Combination strategies have been explored to enhance reovirus efficacy. Co-treatment with GSK2606414, a pERK inhibitor, improved reovirus-induced tumor cell killing in HNSCC (39). Similarly, histone deacetylase inhibitors (HDACi) have been shown to enhance reovirus entry and augment T cell-mediated antitumor responses when combined with Reolysin (a clinical-grade reovirus) (22). Since HDACs mediate histone deacetylation and gene silencing, their inhibitors may also relieve transcriptional repression contributing to oncogenesis (40).

### 3.5 Measles virus

Resistance to ICIs is increasingly challenged in HNSCC, and pre-existing immunity to viral vectors reduces the efficacy of OVs. MV, while common in human populations, is engineered to overcome the problem (41). The attenuated Edmonston lineage measles virus (MV-EDM) preferentially targets tumor cells via the CD46 receptor, which is overexpressed on many cancer types, thus enhancing tumor selectivity (41). Additionally, the MV-EDM strain has been modified to express the sodium iodide symporter (NIS) gene, enabling cancer cells to accumulate radioactive isotopes. When combined with therapeutic radioisotopes such as ¹³¹I, the approach amplifies tumor-specific cytotoxicity (41). The combination strategy has shown strong preclinical efficacy in both HNSCC and sarcoma models (42, 43). The role of MV-NIS in patients with recurrent or metastatic HNSCC is currently being evaluated in one Phase I clinical trial (26). Notably, MV exhibited a favorable safety profile with no dose-limiting toxicities and interacted with host cells via multiple receptors (9) (**Table 3**).

**Table 3:** The viral characterization used in clinical trials of HNSCC.

Virus species	Trial name	Year	Country	Status	Trial type	Virus name
HSV-1	NCT01017185	2009-2015	USA	Completed	Phase I	HF10
	NCT01161498	2011	Multiple	Terminated	Phase III	T-VEC
	NCT02428036	2015-2017	Japan	Completed	Phase I	HF10
	NCT02626000	2016-2020	USA	Completed	Phase I	T-VEC
Adenovirus	–	2001	Multiple	Completed	Phase II	ONYX-015
	Xia ZJ 2	2004	China	Completed	Phase III	H101
	NCT04685499	2021-2022	USA	Terminated	Phase II	OBP-301
VACA	NCT01584284	2012-2015	USA	Completed	Phase I	GL-ONC1
Paramyxovirus	NCT01846091	2013-2019	USA	Completed	Phase I	MV-NIS

### 3.6 Vesicular stomatitis virus

VSV is a non-pathogenic, enveloped RNA virus with robust oncolytic capabilities (44). Human VSV infections are typically asymptomatic. The favorable features of VSV include broad tropism, high lytic ability, rapid replication, and genetic malleability (45–47). VSV induces endoplasmic reticulum (ER) stress in cancer cells, triggering apoptosis, further enhancing its oncolytic efficacy (48). Engineered VSV strains expressing type I interferons (VSV-IFN-β) exploit the antiviral response: while greatly reducing the spread in normal cells, IFN expression paradoxically enhances tumor selectivity and immune activation (49, 50). In HNSCC, recombinant VSV expressing a mutant Newcastle disease virus (NDV) fusion protein rVSV-NDV/F(L289A) induced extensive syncytium formation, accelerating viral propagation and improving therapeutic efficacy (51). These findings suggest potential synergy between VSV and fusogenic proteins, warranting further investigation.

### 3.7 Newcastle disease virus

NDV, a single-stranded RNA virus of avian origin, causes only mild flu-like symptoms in humans, and replicates in the cytoplasm without integrating into host DNA, minimizing the risk of insertional mutagenesis (52). The virus has emerged as a promising OV candidate due to its selective replication in tumor cells and low toxicity in normal tissues (53).

Since many HNSCC lesions are superficial or anatomically accessible, local intratumoral administration of NDV is feasible (54). Repeated local injections also showed some antitumor effect in preclinical models. The structure and immune characteristics of the oral mucosa may influence how viruses spread locally and how much toxicity they cause. Therefore, mucosal or lesion-directed delivery strategies deserve further study in HNSCC (55). In HNSCC models, fusogenic NDV(F3aa)-GFP had selective oncolytic activity. A single intratumoral injection of NDV(F3aa)-GFP decreased tumor burden without clear systemic toxicity; thus, it supports further study of NDV-based therapies for accessible HNSCC tumors (53) (**Figure 2**).

Taken together, current OV platforms differ substantially in viral structure, replication kinetics, tissue tropism, immune activation, transgene capacity, and safety profile. Therefore, no single OV backbone is likely to be suitable for all patients with HNSCC. Selection of an OV platform should consider tumor subtype, HPV status, immune phenotype, anatomical accessibility, route of administration, and the intended combination regimen. The next section summarizes the clinical and translational evidence for OV-based combination strategies in HNSCC.

## 4 Clinical and translational progress of OV-based combination therapy in HNSCC

Clinical results demonstrate good safety and therapeutic efficacy in patients with late-stage HNSCC, while OVT was combined with radiotherapy and chemotherapy and so on.

### 4.1 Surgical treatment

Surgery is still a very important part of curative treatment for patients who have resectable HNSCC, especially for tumors of the oral cavity and some specific types of locally advanced tumors (2). Although organ-preserving options exist for certain patients with late-stage HNSCC, many cases require surgery followed by postoperative radiotherapy or chemoradiotherapy. Postoperative recurrence continues to be a major clinical problem that might result from microscopic residual disease at or beyond the surgical margin. Also, perioperative tissue damage, inflammation, and temporary immune suppression could favor residual tumor cell survival and metastatic progression (56, 57). These considerations offer justification for testing OVT during perioperative or postoperative settings. VACV-based platforms are of interest because they can induce direct tumor cell killing as well as local immune activation. Preclinical models showed that engineered IL-12-expressing VACV strains such as VVΔTKΔN1L-m/hIL12 improved antitumor immunity and decreased post-operative recurrence (16). The above results support further research on OV-based treatments as an adjunct to surgery, but the optimal timing, route of administration, and safety profile for HNSCC are yet to be determined.

### 4.2 Radiotherapy

Radiotherapy is also an essential part of HNSCC therapy and is used together with surgery, chemotherapy or immunotherapy according to different tumor sites and stages as well as HPV status. Patients with HPV-positive disease tend to respond better to radiotherapy compared to those with HPV-negative tumors; some prior reports indicated that 10-year absolute survival was improved for HPV-positive patients treated by radiotherapy-based regimens (58, 59). On the contrary, HPV-negative HNSCC is more closely associated with smoking exposure, a high number of mutations, and frequent TP53 alterations which could cause resistance against radiation and poor outcomes in clinical settings (60, 61). These differences provide a rationale for combining radiotherapy with OVs, particularly for patients with treatment-resistant or HPV-negative HNSCC. A lot of preclinical studies have been done on this approach too. In a human HNSCC xenograft model, a conditionally replicating Ad-TIMP-2 virus was combined with radiotherapy and cisplatin (62). TIMP-2 can block extracellular matrix degradation, angiogenesis and metastatic progression via both MMP dependent and independent ways (63). In this case, adenoviral replication caused tumor cell death while radiotherapy and cisplatin added DNA-damage stress on the tumor cells. The combination treatment produced greater suppression of the tumor than single-agent treatment, suggesting that further exploration into Ad-TIMP-2-based combination therapies for HNSCC should occur (62). And oncolytic vaccinia virus plus radiotherapy has shown stronger inhibition of tumor growth than either treatment alone in preclinical models (64).

A phase I clinical trial showed that the combination of Gendicine (a recombinant adenovirus expressing p53) and radiotherapy had a significant synergistic effect, which improved the tumor-targeted replication of adenovirus (65). OBP-301 is a telomerase-specific oncolytic adenovirus; it increases the effectiveness of radiation therapy by degrading the MRN complex, inducing apoptosis, and overcoming radio resistance in HNSCC. Early clinical trials confirmed its safety as well as compatibility with chemotherapy agents such as cisplatin and paclitaxel (66, 67).

### 4.3 Chemotherapy

Late-stage HNSCC responds more favorably to concurrent chemotherapy and radiotherapy than to radiotherapy alone (68, 69). Numerous OVs, when combined with chemotherapy, have shown significant therapeutic effects in Phase I, II, and III trials, enhancing the objective response rates (ORRs) of patients.

H101, an E1B-deleted oncolytic Ads, has exhibited efficacy in treating nasopharyngeal carcinoma when combined with chemotherapy (65). Recent trials have further underscored the crucial role of cisplatin in treating HPV-positive HNSCC (70). When combined with 5-fluorouracil (5-FU) and oncolytic Ads, cisplatin enhances tumor cell targeting, making it a cornerstone chemotherapeutic agent in HNSCC. In a phase III clinical study, H101 combined with cisplatin plus 5-fluorouracil (PF) was compared with PF alone, and the results showed that the combination therapy had significant efficacy in HNSCC and esophageal squamous cell carcinoma, with good patient tolerability (65, 71).

An oncolytic MV engineered to target EGFR and carry the cytosine deaminase/uridine phosphorylase transferase (CD/UPRT) genes, when combined with 5-FC treatment, has exhibited significant anti-tumor effects, presenting a promising therapeutic strategy for HNSCC (43). Moreover, combining oncolytic HSV-1 with bortezomib has shown significant cytotoxic effects, leading to reduced tumor size compared to bortezomib monotherapy (72).

### 4.4 Integration with immunotherapy: a promising strategy

Cetuximab, a monoclonal antibody targeting EGFR, is frequently combined with radiotherapy for late-stage HNSCC treatment (73). The oncolytic adenovirus Ad5.pK7-D24, when used in combination with cetuximab, cisplatin, and 5-fluorouracil, was found to exhibit anti-tumor ability in HNSCC cell lines. The combination of Ad5.pK7-D24 with chemoradiotherapy and cetuximab shows promising prospects for HNSCC treatment, though further clinical trials are needed (74).

The most promising clinical outcomes have emerged from trials combining OVs with ICIs. ICIs have significantly improved survival in some HNSCC patients (75, 76). However, only about 20% of patients show substantial benefits from ICIs (77), and even those who initially respond may develop resistance (78). A multicenter Phase Ib/III trial investigated intratumoral T-VEC in combination with pembrolizumab in patients with platinum-refractory HNSCC. While early results indicated enhanced immune infiltration and tolerability, the study was not powered for efficacy outcomes, and biomarker correlates were underexplored (79). Resistance to ICIs is often associated with Treg infiltration and immunosuppressive cytokines such as TGF-β within the TME. Engineered CPXV strains (VVdnTGFβmm) combined with ICIs may overcome the resistance by deleting Treg cells and reducing TGF-β signaling (33). Furthermore, CPXV has been shown to enhance the efficacy of various immune modulation therapies, particularly those hindered by TGF-β–mediated immunosuppression (80). Additionally, recombinant Ads engineered to express IL-2 and TNF-α exhibited synergy when combined with ICIs, offering a promising strategy for improving clinical responses in ICI-resistant or refractory HNSCC patients (76).

**Figure 3:**
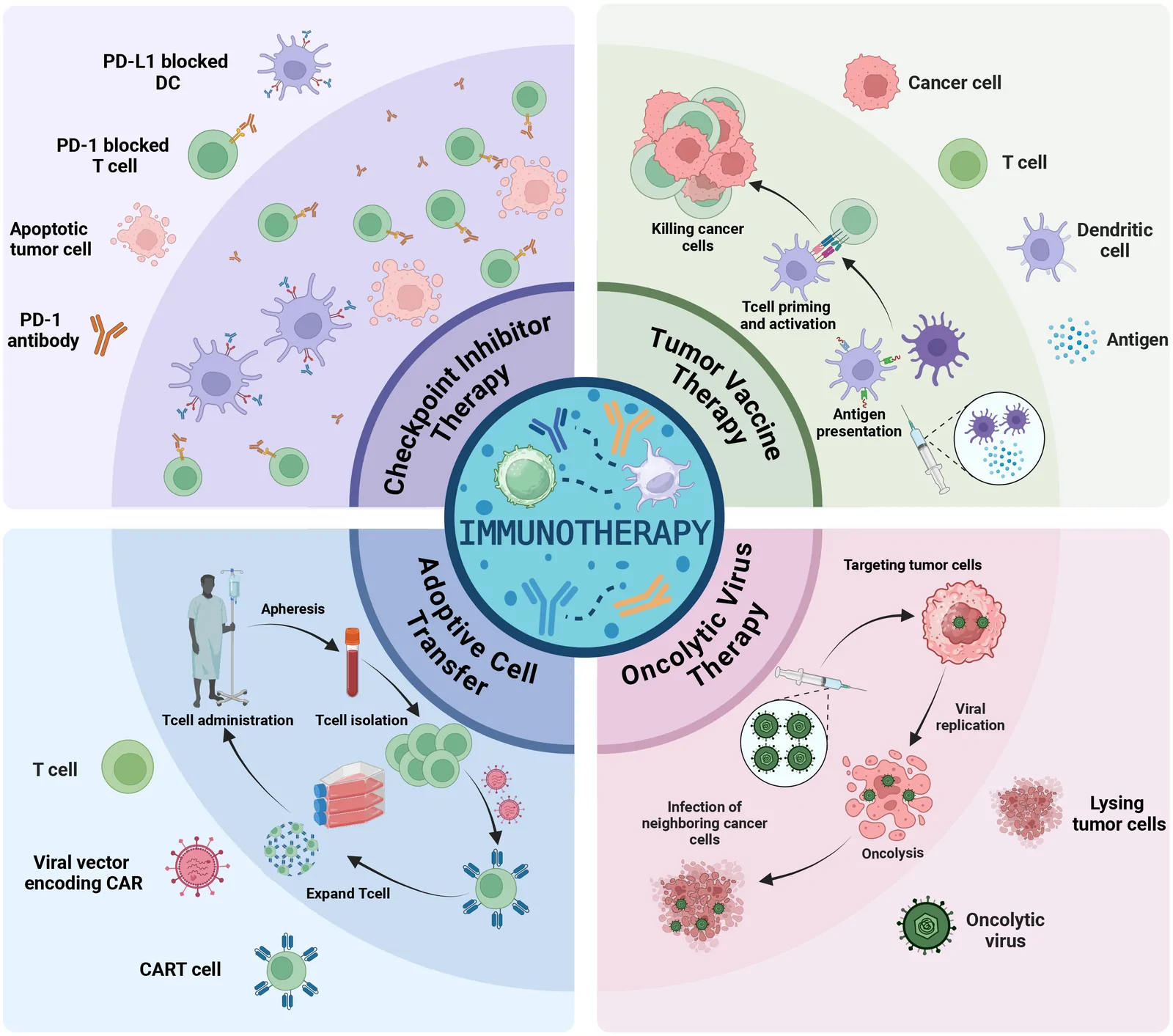
Immunotherapeutic strategies for HNSCC treatment. Four main immunotherapeutic strategies have been developed for HNSCC: (1) checkpoint inhibitor. (2) tumor vaccine therapy. (3) adoptive cell transfer. (4) oncolytic virus therapy. The figure was created using BioRender (https://biorender.com/).

Combining OVs with ICIs, adoptive cell therapies, radiotherapy, or targeted agents may yield synergistic outcomes. These combinations aim to counteract the immunologically “cold” TME that typifies many HPV-negative HNSCC tumors (**Figure 3**).

## 5 Barriers to translation

OVs can be versatile treatments for HNSCC but many obstacles still exist which hinder the successful transition from the laboratory to the clinic. Challenges include immune evasion, physical/anatomical limitations on delivery, variable viral tropism, and genetic instability of viral constructs. Understanding these limitations is important for OV engineering and clinical trial design.

### 5.1 Physical, anatomical, and systemic delivery barriers

Although many HNSCC lesions are either on the surface or reachable by direct injection, it remains hard to spread the virus uniformly throughout the tumor. Extracellular matrix and high interstitial fluid pressure as well as intratumoral compartmentalization could hinder the spread of viruses which cause different parts of the tumors to get different amounts of exposure. Also, incorrect positioning of the needle and leakage during administration might lower the effective viral quantity that reaches the cancerous cells and raises neighboring normal tissue’s contact (81, 82). Systemic administration has its own set of obstacles. Circulating neutralizing antibodies and quick immune clearance can decrease the bioavailability of OVs, especially for commonly encountered viral backbones such as adenovirus and measles virus. This is likely even more true for patients who’ve been exposed before to wild-type strains or gotten vaccinated; they already have some antiviral immunity that would make intravenous delivery less efficient. So, we need to improve tumor localization while limiting off-target distribution. Ultrasound-guided microbubble delivery, polymeric hydrogels, and immune-cell-based carrier systems may increase local retention, protect the virus from being cleared by the immune system, or help move the virus into the tumor site. But most of the evidence is still preclinical, and these kinds of delivery methods haven’t been tested in big HNSCC-specific clinical trials (83). The creation of intratumoral delivery devices, immune-shielding biomaterials, and cell carriers is very important in the complicated head and neck area.

Importantly, the accessibility of many primary HNSCC lesions does not ensure adequate control of systemic disease. The patients with recurrent or metastatic HNSCC are often present with multiple nodal, distant, or occult lesions that cannot be fully treated by intratumoral injections. Although local OV administration can elicit systemic antitumor immune responses, immunity generated against a single primary or locoregional lesion may not provide uniform protection across all metastatic sites. HNSCC is characterized by substantial spatial and temporal heterogeneity, with paired-sample and single-cell studies revealing genomic evolution, changes in neoantigen profiles, and distinct immune microenvironments among primary, recurrent, and metastatic lesions (84, 85). Such interlesional differences may influence viral entry and replication, susceptibility to direct oncolysis, and the effectiveness of immune-mediated tumor clearance. Therefore, the development of repeatable systemic OV delivery is essential for achieving adequate viral exposure across anatomically dispersed and biologically heterogeneous disease sites.

### 5.2 Immunologic resistance within the TME

The immunosuppressive TME of HNSCC is one of the primary obstacles to effective oncolytic virotherapy. In HPV-negative HNSCC, immune exclusion, increased Treg infiltration, and elevated levels of suppressive cytokines (e.g., TGF-β, IL-10) create a tumor environment that blocks immune activation even after OV-mediated antigen release (83). Conversely, while HPV-positive tumors are more immunogenic, their response to OVT is often constrained by checkpoint overexpression and stromal shielding. Furthermore, Previous studies found that OV efficacy was reduced due to the recruitment of MDSCs, which inhibited the activity of virus-induced cytotoxic T lymphocytes (86).

### 5.3 Genetic instability and payload loss

Engineered OVs frequently carry therapeutic transgenes, such as cytokines, checkpoint inhibitors, and immune agonists—which are subject to deletion under replicative stress (80). In some cases, serial replication in tumor cells may result in the loss of transgene integrity, undermining the immune-stimulatory function of the virus while retaining replicative potential, reducing therapeutic efficacy, and raising biosafety concerns related to uncontrolled viral spread or recombination (87–90). To address this, researchers have incorporated genetic “stabilizers” such as scaffold domains, self-cleaving peptides (e.g., P2A), and insulators, even though long-term transgene retention remains an unresolved challenge in high-multiplicity infection settings.

### 5.4 Tumor heterogeneity and lack of targeting specificity

HNSCC is highly heterogeneous at both genomic and immune levels, which affects viral receptor expression, antiviral response signaling (e.g., IFN pathway defects), and the local cytokines that determine viral replication and immunogenicity (91). For example, HSV-1 entry is dependent on nectin-1 or HVEM, both of which are variably expressed across HNSCC subtypes (92).

Genetic engineering strategies such as transcriptional targeting (e.g., using tumor-specific promoters, such as hTERT), miRNA-responsive control circuits, and fiber-modified viral capsids enhance selectivity, but often require extensive validation in context-specific models.

## 6 Engineering solutions and novel strategies

To achieve the full therapeutic potential of OVs in HNSCC, we have seen more improvements in synthetic virology and immunoengineering resulting in even better viral platforms (93). In simple terms, these strategies are designed to help OVs find tumor cells more accurately, spread more efficiently inside tumors, activate stronger immune responses, and keep viral replication under better control.

### 6.1 Enhancing intratumoral spread and overcoming ECM barriers

The limited penetration of the tumor is a big problem for OV treatment, especially if there is abundant stroma or fibrosis, or areas without much oxygen. In order to enhance intratumoral spread, some OVs were modified so they would express matrix modifying enzymes. For instance, hyaluronidase PH20 which degrades extracellular matrix components allows viral diffusion and immune cell infiltration (45, 94). ICOVIR17 is an example of this approach and showed better intratumoral dispersion and antitumor effects by targeting stromal barriers in preclinical models (95). Other methods aim to increase local viral retention or promote cell-to-cell spread. Ad-based vectors have been combined with immunomodulatory agents, antibodies, or biomaterials to improve local delivery. SELP hydrogel can release adenovirus continuously and it has been reported to improve anti-tumor activity in experimental models (96, 97). Fusogenic viral engineering gives us another method to deal with limited extracellular diffusion. HSV strains carrying mutations in glycoprotein B or D can cause syncytium formation, enabling lateral viral spread by direct cell-cell fusion (98).

### 6.2 Engineering strategies for efficient systemic OV delivery

The immune response triggered in a primary HNSCC tumor may not control metastases. The systemic delivery of OVs may directly bring them to occult or distant lesions. Once in the bloodstream, however, OVs can be neutralized by antibodies or/and complement or removed by antigen-presenting cells and macrophages before reaching tumor tissue. Strategies under investigation include cell-mediated, protein-mediated, and nanoparticle-based delivery (99–102). For the cell-based approach, Zheng et al. used MV infected CAR-T and TCR-T cells to carry the virus to antigen-expressing solid tumors. The treatment was found to induce tumor cell autosis and bystander killing of antigen-negative cells, reducing the antigen-escape-mediated resistance in one preclinical tumor model (103). The clinical study about the intravenous injection of VCN-01 has been completed in HNSCC patients with recurrent or metastatic disease. VCN-01 was detected in both blood and tumor tissue, together with the evidence of viral replication, extracellular matrix remodeling and increased CD8 expression. Sequential dosing was better tolerated than concomitant dosing (104). Recently, Ban et al. packaged an oncolytic adenovirus in engineered bacterial outer membrane vesicles with a calcium phosphate coating. After intravenous administration, the nanoparticle-based delivery reduced viral clearance and favored tumor rather than liver accumulation and increased intratumoral viral replication and limited local recurrence and distant tumor growth (105). Newer delivery platforms, particularly cell carriers and biomaterial-based shielding have been studied, however, remain largely uncertain (106). Our group also investigate how reliably systemically administered OVs reach, replicate in, and act against individual primary and metastatic HNSCC lesions.

### 6.3 Receptor retargeting and capsid modification

Tumor specificity is improved by altering viral entry tropism. Fiber-modified adenoviruses have been engineered to recognize integrins (RGD motifs) or CD46, receptors that are overexpressed on HNSCC tumor cells, enhancing infection efficiency while bypassing receptors poorly expressed in target tumors (107). Similarly, measles virus strains retargeted to bind EGFR or ICAM-1 were shown to improve selectivity for epithelial malignancies (108). These modifications expand the repertoire of tumor types that are effectively targeted and allow for systemic administration in cases where intratumoral delivery is not feasible.

### 6.4 Payload engineering: Immuno-stimulatory and metabolic modulators

Modern OVs are frequently armed with transgenes that modulate the TME. Cytokines have been widely used in engineered OVs; however, a novel cytokine combination (such as IL-15/IL-12) was found to promote DC maturation, T cell activation, and NK cell recruitment more efficiently (109). Recently, some metabolic modifiers (such as leptin or arginase inhibitors) were revealed to reprogram tumor metabolism to favor immune activation, while they were engineered into OVs (110). These strategies may create a multi-hit immunological effect, turning immunologically “cold” HNSCC into “hot” inflamed, and T cell–infiltrated environments.

### 6.5 Influence of tumor plasticity on OVT

Tumor plasticity may reshape both intrinsic antiviral immunity and tumor immunogenicity in HNSCC by altering the expression of viral entry receptors, antiviral signaling, neoantigen repertoires, HLA class I-mediated antigen presentation, and immune-checkpoint ligands (85, 111, 112). Even though direct evidence that these changes affect OV infection and replication in HNSCC remains limited, for other kinds of cancer, Some of research study about these have been performed. OVT strategies may account for dynamic tumor-cell states in HNSCC. Combining OVs with immune checkpoint blockade prevented immune escape driven by state-dependent changes in PD-L1 and other checkpoint-associated ligands, such as CD80 and CD155. Alternatively, engineering OVs to express checkpoint inhibitors provided more localized checkpoint blockade and potentially reduced systemic exposure (111, 113). Longitudinal single-cell profiling of primary, recurrent, and metastatic lesions may track treatment-induced state transitions and inform the selection of viral backbones, therapeutic payloads, and combination regimens (85). In summary, increased evidence arises showing that tumor plasticity plays a major role in governing intrinsic immunity of solid tumors like HNSCC.

## 7 Perspectives and outlook

**Figure 4:**
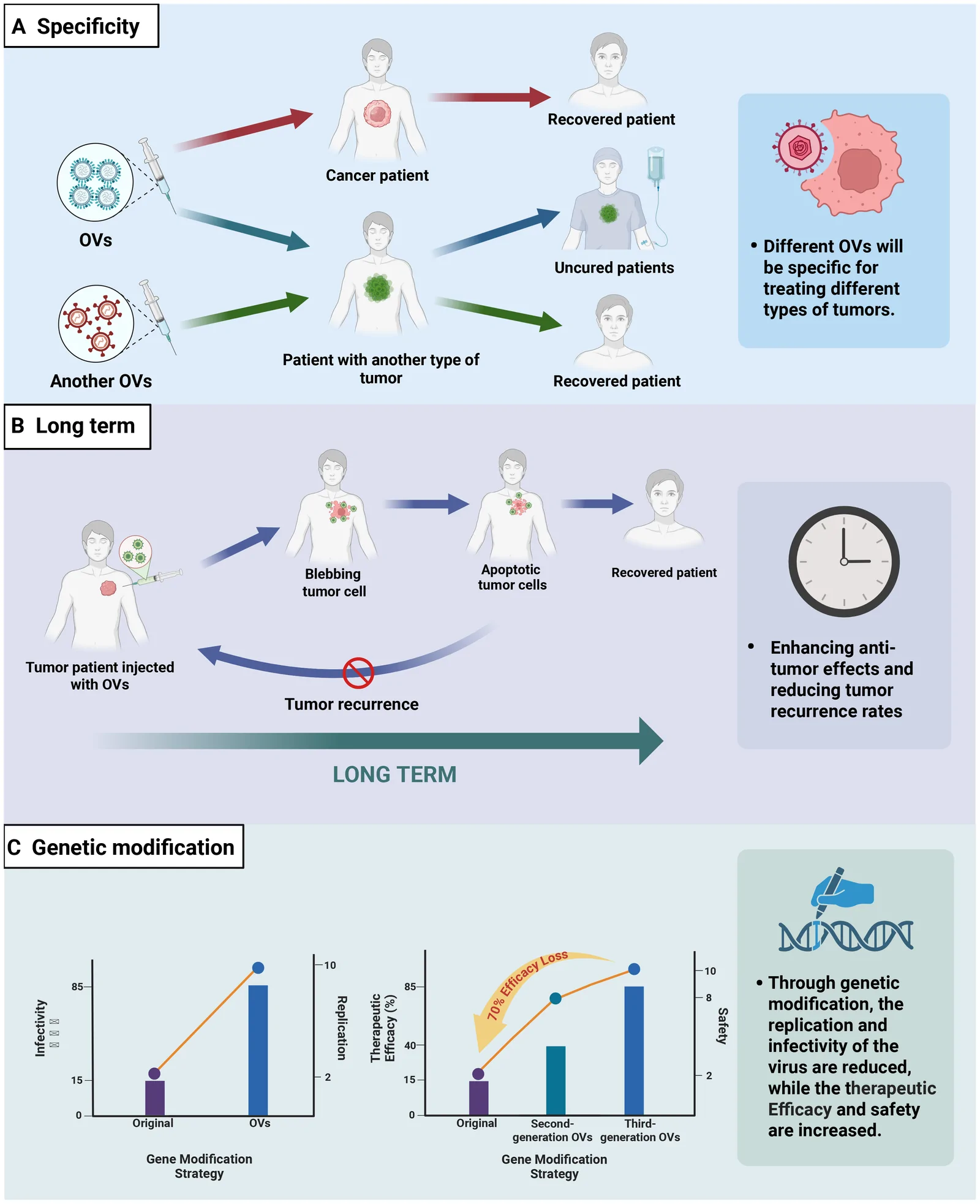
Perspectives and outlook of OVs. **(A)** Specificity: Genetically modified OVs enhance specificity and therapeutic efficacy. **(B)** Long-term effects: The sustained anti-tumor effects and reduced recurrence with OVT. **(C)** Genetic modification: Genetic modifications improve replication, safety, and efficacy of OVs for targeted tumor therapy. The figure was created using BioRender (https://biorender.com/).

HNSCC is often diagnosed at an advanced stage and is characterized by immune escape, treatment resistance, and limited responses to single-agent therapy. These features provide a biological rationale for testing OVs in this disease setting. However, the clinical value of OVT will depend on whether several practical and mechanistic barriers can be addressed. Future development should focus on more precise vector design and more selective clinical deployment (**Figure 4**). This includes selecting viral backbones according to tumor receptor expression, interferon signaling status, HPV status, and immune phenotype; using biomarkers such as TGF-β pathway activity to identify patients who may benefit from specific engineered viruses; and monitoring intratumoral viral replication with reporter genes or theranostic imaging systems, including NIS-expressing viruses. In addition, Zhao et al. developed an NDV-based treatment strategy for refractory cancers based on hyperacute immune rejection, providing another example of how viral platforms may be adapted for gene- and immune-based cancer therapy (87).

From a regulatory and translational perspective, OVs also need more standardized efficacy endpoints, long-term safety assessment and scalable manufacturing processes. Differences in viral backbones, transgenes, delivery routes, and combination regimens make it difficult to compare results across studies and complicate regulatory evaluation. So future trials should include harmonized design elements, immune-monitoring assays, and correlative endpoints that link viral replication with immune activation as well as clinical response. For HNSCC, OVs are unlikely to replace current treatments in the near term. More realistically, they may become part of multimodal therapy when selected according to tumor biology and combined with radiotherapy, chemotherapy, or immune checkpoint blockade.
